# Emerging Cognitive Intervention Technologies to Meet the Needs of an Aging Population: A Systematic Review

**DOI:** 10.3389/fnagi.2019.00291

**Published:** 2019-10-24

**Authors:** Fady Alnajjar, Sumayya Khalid, Alistair A. Vogan, Shingo Shimoda, Rui Nouchi, Ryuta Kawashima

**Affiliations:** ^1^College of Information Technology, United Arab Emirates University, Al Ain, United Arab Emirates; ^2^Intelligent Behavior Control Unit, CBS-TOYOTA Collaboration Center, RIKEN, Nagoya, Japan; ^3^Institute of Development, Aging and Cancer (IDAC), Tohoku University, Sendai, Japan

**Keywords:** cognitive impairment, cognitive training, computerized cognitive training, socially assistive robots, robotics for elderly

## Abstract

**Background:** Cognitive training helps to promote healthy aging and ease activities of daily living for older adults. Recently, experiments have been conducted using robots to perform this cognitive training.

**Methods:** A review was conducted to examine the effects of computer-based cognitive interventions for older adults who were either healthy or experiencing mild cognitive impairment (MCI). A second study also examined the evolution of socially assistive robots (SAR) and their effectiveness at administering cognitive training for older adults.

**Results:** Eighty-one studies published between 2009 and 2019 were identified for review, 56 of which focused on computerized cognitive training (CCT) while 25 examined the use of robotics. Twenty-four of the 56 CCT studies met the inclusion criteria. These were further classified into two groups: studies which used self-designed programs, and studies using commercially available ones. Of the 25 studies examining the use of robotics in cognitive intervention 7 met the inclusion criteria. Review shows that CCT improves cognitive function but that robots are more effective tools for improving cognition.

**Conclusion:** It can be concluded that CCT is beneficial for older adults and though there are drawbacks to this approach they are overcome by the introduction of robots into the training process. Culture, language, and socio-economic considerations vis-a-vis robot design and training methodology should be included in future research.

## Introduction

In recent decades, the world has witnessed measures of poverty drop while, on average, those of education, income, quality of life, and life expectation have risen significantly (Pinker, 2018). Indeed, on many measures there is reason for optimism. However, a quick study of the demographics indicates a rapidly aging global population^1^. It is during this period that many begin to experience the challenges of performing simple daily self-care and other independent living activities (McColl et al., 2013). This is because, in the later years, cognitive functions such as working memory have been found to diminish, while the prevalence of various diseases and disorders, including age-related dementia and Alzheimer's Disease, grows (Bozoki et al., 2013). In fact, dementia is one of the main reasons for the increased dependency of older people since it results in the deterioration of those specific cognitive functions needed in daily life (McColl et al., 2013). This deterioration is manifested in symptoms such as loss of memory, problems of orientation, depression, behavioral changes, and impaired communication skills. According to recent findings, each year over 9.9 million new cases of dementia are identified worldwide; this suggests a new case emerging every 3.2 s^2^ At this rate, by 2050, those experiencing dementia will have reached an alarming 131.5 million^2^. The financial impact of this has the potential to be overwhelming. While the present economic worldwide cost of dementia is approximately 818 billion US dollars, in only a few years it is expected to climb to a trillion-dollar challenge^2^. This, too, applies acutely to the United Arab Emirates (UAE). At present, the UAE possesses a relatively young population; however, in the coming years this is projected to change. According to the United Nations World Economic Situation and Prospects 2010 Report, by 2050 there will be a substantial rise in the population of those aged 60 or over, from 2.4% to an alarming 27%^1^. For healthcare systems to fully-prepare themselves to meet this new reality significant innovations will need to be explored. Since no effective treatment or cure for dementia exists, an increased effort is being made to establish the efficacy of non-pharmaceutical strategies. One of these strategies is targeted cognitive training for older adults which may lead to prevention of dementia or delay of its onset (Brinke et al., 2018).

Cognitive training has been shown to maintain, or even improve, cognitive function for elderly (Kueider et al., 2012). In the past two decades this form of training has gained popularity. Studies have demonstrated its effectiveness in improving memory, attention and cognitive skills (Willis et al., 2006; Mowszowski et al., 2010; Kelly et al., 2014; Rebok et al., 2014). One randomized trial using cognitive training found diminishment in the decline of instrumental activities of daily living (IADL), thus leading to prevention and reduction of further risk of developing functional decline in elderly (Rebok et al., 2014).

Cognitive training involves a well-structured practice of complex mental exercises. Training can be given in multiple ways; it can be process-based, comprising repetitive training on specific tasks, or more strategic, individualized intervention based on memory formation strategies (Walton et al., 2015). However, there are hurdles to its widespread implementation. The traditional method of cognitive training requires a trained instructor, for example. This necessitates face-to-face interaction, which entails a meeting location, the coordination of schedules and travel time. Additionally, training can be very expensive since trainers usually charge by the hour; there is also the added cost of equipment and materials. Furthermore, not all elderly individuals are comfortable in traveling regularly to a meeting location. In fact, some older adults may be home-bound, live in an assisted living or nursing home facility, or may simply not be able to easily access transportation (Kueider et al., 2012). As a result, it becomes difficult to take part in these programs with regularity.

Recent informational technological advancements which potentially alleviate this problem have made their way into healthcare, and now play a significant role in cognitive training. Computer-based cognitive training (CCT) has been found to be easier to implement since it is cost-effective, can be accessed from anywhere and at any time, and can be performed from the comfort of the user's home (Kueider et al., 2012). Also, it can be customized according to specific needs of individuals. Moreover, CCT provides real-time performance assessment and feedback, and allows for the adjustment of application difficulty level accordingly. There are three approaches to impart CCT: (1) brain-training programs, (2) working memory training programs, and (3) video game training programs (Boot and Kramer, 2014). Computer and video games are designed to be fun and exciting. This serves to motivate users to maintain engagement throughout the training program. However, while there are many cognitive training products in the market, there is still a lack of evidence supporting their effectiveness at imparting cognitive training with significant improvement in cognitive function (Kueider et al., 2012). One study, a meta analytical review concluded that a commercially available computer-based training program for working memory skills was only able to have short-term specific training effects and did not generalize to “real world” cognitive skills, which raises question regarding the methodological approach or theoretical support for the current available training mechanisms (Melby-Lervåg et al., 2016).

To provide effective care or to improve the efficacy of the training program, it is important that the methods used should be comfortable for users. For example, the elderly may feel more at ease when training is conducted in their native tongue and when it is developed with an orientation to their individual culture. The consequence of this will be that training is more impactful and enjoyable since the user will be more greatly motivated to engage within each session. Furthermore, with increasing research being conducted on interventions for age-related cognitive impairment, it is important to understand and distinguish the effectiveness of various methodologies used. The effectiveness of any individual methodology may be determined, for practical purposes, by the extent to which a transfer effect is produced. The transfer effect, in this case, would refer to the effect that the knowledge or abilities acquired in one area might have on the knowledge acquisition in other areas. From this understanding, the transfer effect can be bifurcated into “near” and “far” transfer effect, with the further distinction that the production of a range of “near” and “far” transfer effects might identify a quality methodology (Nouchi and Kawashima, 2014). While short-term cognitive training has been demonstrated to produce a limited and temporary effect, training conducted regularly and with vigor over an extended period of time can have a sustained meaningful impact (Tapus and Vieru, 2013). Unfortunately, the healthcare facilities are already under pressure with a shortage of staff and space. It is very challenging to provide a customized setup for each individual, according to their specific requirements (Tapus and Vieru, 2013).

Intelligent robotic systems have been designed for human-robot interaction (HRI). HRI is a field of study dedicated to understanding, designing and evaluating robotic systems for use by or with humans. It's a communication link between human and robots^3^ As per Wikipedia the purpose of HRI is to model human expectations, regarding robotic interaction, to aid in robot design and algorithm development, which can allow more natural and effective interaction between human and robots.

Newly developed robotic systems have evolved considerably and can now be effectively used to provide that individualized care to the elderly, and from the comfort of the user's home (McColl et al., 2013). Additionally, socially interactive robot may have a tremendous impact on overall cognitive and social well-being. Developing a more human-like social robot with natural gestures and speech can engage a user and more fully support them as they carry out their exercises (Tapus and Vieru, 2013). Robots such as these could be programmed to help users choose from a variety of exercises and could motivate them along the way by giving applause, praises, or encouraging feedback during the training. Additionally, social assistive robots (SAR) have been shown to provide a companionship which improves user engagement in activities. This plays a powerful role in cognitive health (Tapus and Vieru, 2013). Furthermore, the specific needs of the user can be met through a customization of the social robot appearance; in particular, they have been designed to resemble pets, such as dogs or seals, producing positive benefits. Robots like Paro, ICat, Albo, and Pearl have been studied for their effect on the elderly, and a positive psychological and social impact has been demonstrated, such as improvement to both the mood and well-being of the users (Broekens et al., 2000). Unfortunately, most interventions have been limited to nursing homes or health facility. Few studies exist which examine the impact of assistive robots on the basic daily activities of the elderly in their own homes.

It seems however, a future socially assistive humanoid robot could help not only with the daily activities of the elderly and by providing company, but also by performing cognitive training with regularity and accuracy. When doing this it would be able to maintain users' scores and learn and adapt continuously to these individuals over time as cognition improved. This adaptive, user-friendly, reliable robot would provide an engaging and motivating customized therapy to users, establishing a life-enriching human-robot relationship.

To make this possible, some system requirements that can be identified are two-way communication, safety, services and assistive functions, therapy and smart situation awareness (Gross et al., 2011). Furthermore, to ensure a good human robot relationship and interaction, we need to make the robot as human-like as possible. The main requirements would be for it to have more appealing, human-like interaction capabilities, demonstrate appropriate social behavior and be able to focus user attention in order to help achieve specific goals (Tapus et al., 2007).

There are a few areas of focus that need to be addressed when designing a humanoid robot: (1) physical appearance, (2) personality, (3) empathy, (4) engagement, (5) adaptation, and (6) transfer (Tapus et al., 2007).

A vast amount of literature exists which explores the effects of cognitive training (both computerized and non-computerized) on the elderly.

This review focuses on the effect of CCT on samples (healthy older adults and healthy older adults with MCI), to delay or prevent onset of dementia. For the purpose of this review, related articles published over the last 10 years were researched to answer the following questions: (1) To what degree has CCT been impactful as a tool for cognitive training of individuals experiencing age-related cognitive decline? (2) Can CCT delay or prevent the onset of dementia and which programs have been found to be most effective? (3) How have robots been used for cognitive training? (4) What challenges have been identified in robot development as they relate to cognitive training of the elderly?

## Methods

To carry out this review, a methodological approach was followed.

### Search Strategy

Databases were searched systematically to identify possible studies for inclusion. The databases were searched using the following keywords: *aging, smart aging, elderly, old, adults, computerized, cognitive, computerized cognitive, training, interactive gaming, cognition, cognitive abilities, video games, trainings, robots, socially assistive robots*. Databases used were *PubMed, Psych Info, SCOPUS, Google Scholar, MEDLINE, and CINAHIL*. We also dug deep into references of some these studies to find out more relevant studies.

### Inclusion and Exclusion Criteria

**Figure 1:**
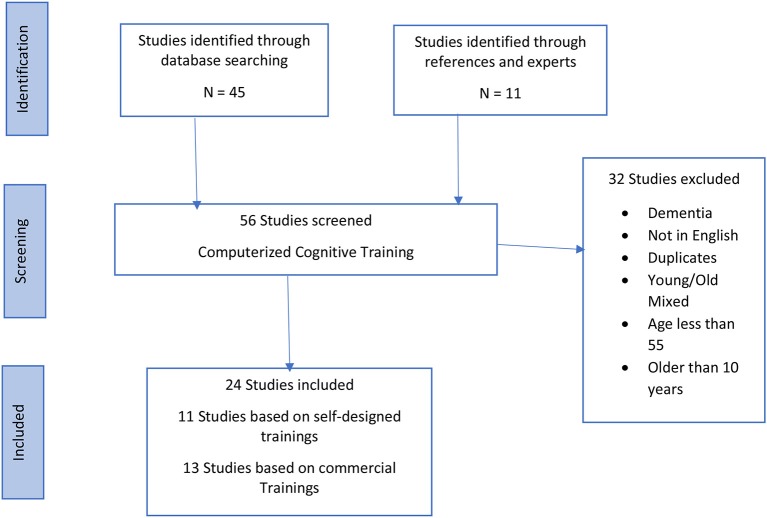
Computerized Cognitive Training (CCT)—flow diagram of search results.

Studies met the inclusion criteria if they: (a) were published in the last 10 years, (b) were in English, (c) were randomized control trials, (d) had a sample of healthy older adults and healthy older adults with mild cognitive impairment, (e) Patients more than 55 years of age [as 3 relevant studies (Ballesteros et al., 2014; Marusic et al., 2016; Zhang et al., 2019) used older adults aged from 55 years old] (f) used only CCTs as intervention (either commercially available or video games). Studies that did not use computer-based trainings were excluded. All studies in which participants had dementia or Alzheimer's disease were excluded. Studies which focused on computerized cognitive training were sorted into two groups, one which used training programs that were specifically designed for the study, the other which used commercially available cognitive training programs, Figure 1. Each study was reviewed and key information (participants, age, type of intervention, cognitive status, and cognitive outcomes) pertaining to study design was extracted.

**Figure 2:**
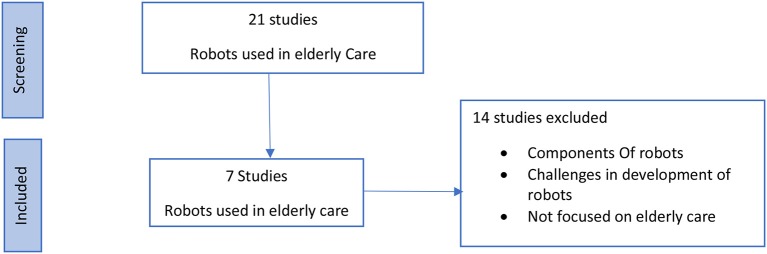
Robots used in elderly care—flow diagram of search results.

In addition, analyzed separately were a few studies, shown in Figure 2, that focused on elderly care using robots. Among these, two types of studies were identified. The first set of studies were based on socially assistive robots, or service type robots, that assisted the elderly in independent living activities. The second set of studies focused on robots which resembled pets, or companion robots, meant to keep users company to mitigate loneliness and depression. Few studies existed which examined the use of robots to impart cognitive training to the elderly. Consequently, studies that were considered eligible for review were those that met a purpose to serve the elderly in any manner. Studies with elderly people with dementia were also included. Excluded, however, were those meant only to gauge the acceptance of robot presence by the elderly, or studies using surgically assistive robots. However, since research examining the effectiveness of using robots for cognitive training is still in its infancy, a time range was not defined for including studies relevant to usage of robotics in elderly care, and the studies possessing participants with dementia were also included for this section.

### Quality Assessment Methodology

Each study was assessed for quality using a modified Delphi list (Verhagen et al., 1998). To improve the assessment quality, the following elements were considered: age range, sample size, and intervention duration. The latter two were considered with the understanding that a smaller sample size and a shorter intervention duration might negatively impact generalizability and reproducibility. Additional considerations were given to a study's limit or extensiveness of the cognitive functions under examination, whether the interventions were conducted at home or at a center, and finally, whether they were supervised or non-supervised. The choice of elements included in the quality assessment was guided by a previously published systematic review conducted by Nouchi et al. (Nouchi and Kawashima, 2014).

## Results

Fifty-six RCTs based on CCT were identified for this review published in the last 10 years. Each study was reviewed and information pertaining to the study design, sample characteristics (e.g., age, cognitive status), cognitive outcomes were extracted. Based on the above-mentioned inclusion and exclusion criteria 24 of the 56 publications related to computerized cognitive training, were eligible for current review. Common reasons for exclusion were: samples having dementia or with participants younger than 55 years of age, duplicate studies, studies not published in English. The 24 studies used in this review focused on different cognitive measures. Table 1 shows the methodological quality of the included studies. Tables 2, 3 summarize the findings of each individual study. Twelve (Barnes et al., 2009; Herrera et al., 2012; Rose et al., 2012; Bozoki et al., 2013; McAvinue et al., 2013; Corbett et al., 2015; Gooding et al., 2015; Marusic et al., 2016; Nouchi et al., 2016; Yeo et al., 2018; Requena and Rebok, 2019; Zhang et al., 2019) studies designed their own CT programs. Twelve (Finn and McDonald, 2011; Peretz et al., 2011; Miller et al., 2013; Strenziok et al., 2013; Ballesteros et al., 2014; Hughes et al., 2014; Hyer et al., 2015; Styliadis et al., 2015; Walton et al., 2015; Lin et al., 2016; Toril et al., 2016; Simon et al., 2018) used a commercially available cognitive training (CT) program based on video game trainings, such as Lumosity, Cog Med, Brain Age etc.

**Table 1:** Scores of methodological qualities.

**References**	**Q1**	**Q2**	**Q3**	**Q4**	**Q5**	**Q6**	**Q7**	**Q8**	**Q9**	**Total Score (max = 9)**
McAvinue et al. (2013)	N	Y	Y	N	N	N	Y	?	Y	4
Yeo et al. (2018)	Y	Y	Y	Y	Y	?	Y	N	Y	7
Bozoki et al. (2013)	N	Y	Y	Y	N	N	Y	Y	Y	6
Corbett et al. (2015)	Y	Y	Y	Y	N	N	Y	Y	Y	7
Rose et al. (2015)	N	Y	Y	N	Y	Y	?	?	Y	5
Nouchi et al. (2016)	N	Y	Y	Y	N	N	N	Y	Y	5
Requena and Rebok (2019)	N	Y	Y	Y	Y,	Y	N	?	Y	6
Zhang et al. (2019)	N	Y	Y	Y	Y	Y	?	?	Y	6
Barnes et al. (2009)	N	Y	Y	Y	N	N	Y	?	Y	5
Marusic et al. (2016)	N	Y	Y	Y	Y	Y	Y	?	Y	7
Herrera et al. (2012)	N	Y	Y	Y	Y	Y	N	N	Y	6
Gooding et al. (2015)	N	Y	Y	Y	Y	?	N	?	Y	5
Hyer et al. (2015)	N	Y	Y	Y	Y	N	Y	?	Y	6
Walton et al. (2015)	N	Y	Y	Y	N	N	Y	Y	Y	6
Toril et al. (2016)	N	Y	Y	Y	Y	Y	Y	?	Y	7
Simon et al. (2018)	N	Y	Y	N	N	N	Y	?	Y	4
Strenziok et al. (2013)	N	N	Y	Y	N	N	N	?	Y	3
Peretz et al. (2011)	Y	Y	Y	Y	N	N	N	Y	Y	6
Finn and McDonald (2011)	N	Y	Y	Y	N	N	Y	?	Y	5
Ballesteros et al. (2014)	N	Y	Y	Y	Y	Y	Y	?	Y	7
Styliadis et al. (2015)	N	Y	Y	Y	?	?	N	N	Y	4
Miller et al. (2013)	N	Y	Y	Y	?	?	Y	?	Y	5
Lin et al. (2016)	N	Y	Y	N	N	N	N	?	Y	3
Hughes et al. (2014)	N	Y	Y	?	N	N	N	?	Y	3

*Q1. Sample size > 199; Q2. Age mentioned; Q3. Duration > 3 weeks; Q4. Cognitive domains > 2; Q5. Supervised intervention; Q6. Carried out at center; Q7. Subjects similar at baseline; Q8. Patients blinded to trial; Q9. Statistically significant*.

*Y, Yes—The study met the criteria. N, No—The study did not meet the criteria. ?, No information*.

**Table 2:** Self-designed cognitive trainings.

**References**	**Type of intervention**	**No of subjects and trial period**	**Cognitive domain focused**	**Findings**	**Limitations**
McAvinue et al. (2013)	•Computerized training task •Control group	36 healthy older subjects Age range: 64–79 years old 5-week training period + a 6-month follow up	•Short-term memory •Working memory	•Improvement in short-term memory, together with transfer of training gains to long-term episode memory tasks •No significant improvement in working memory	•A small sample size •Lack of inclusion of a measure of visuo-spatial short-term or working memory •Non-adaptive version of the training program for control group
Yeo et al. (2018)	•Cognitive training system, BRAINMEM	240 healthy participants Age range: 60–80 years old 24 sessions over 8 weeks and three-monthly booster sessions	•Attention •Working memory •Delayed recall	•No significant differences in overall cognitive performance post-intervention between subjects	•Lack of a sham control •Unbiased testing of effect sustainability of the training not done •Lack of generalizability
Bozoki et al. (2013)	•Online games designed for the program •Active group only	60 Healthy older subjects Age range: 60–80 years old 6 weeks	•Visual attention •Working memory •Processing Speed •Reasoning	•No effects, only improvements on games	•A small sample size; a short-term trial •No control group •Low program intensity
Corbett et al. (2015)	•Problem-solving cognitive training (ReaCT) •General Cognitive Training (GCT) •A control treatment •Group	2,192 healthy older subjects; Age mean: 65 years old 6 months	•Reasoning •Problem solving •Attention •Memory •Visuospatial ability	•Improved cognition, particularly the reasoning skills, evident from week 6	•Only people who could access computer were included into the trial •Only people with higher levels of education; retention strategies need to be improved
Rose et al. (2012)	•Virtual Week Training •Program •Active Control •Group (ACG)	59 healthy older subjects Age mean: 67.4 years old 1 month 12 sessions, each 1 h long	•Prospective memory	•Improved prospective memory •Transfer to real-world settings, reflected in participants' daily activities	•A small sample size •A short-term trial period •A lack of effective strategies used by participants
Nouchi et al. (2016)	•Processing Speed Training Game (PSTG) •Knowledge and Quiz Training Game (KQTG) •Active control group	72 healthy older adults Age range: 60 years old or more 4 weeks	•Processing speed •Reasoning •Short term memory •Working memory •Episodic memory	•PSTG had a small improvement in processing speed, inhibition and depressive mood •No improved performance in reasoning, shifting, short term/working memory, and episodic memory	•Short-term training period •No follow-up assessment •A small effect size
Requena and Rebok (2019)	•Experimental control group •G1—Training with Lumosity •G2—Training with paper and pencil	54 healthy older adults •Age range: 65 years and older 32 sessions held weekly during the months of October to May during the years 2015–2017	•Attention •Memory •Psychological well-being	•No differences in the psychological well-being in either groups •Significant difference in attention, everyday memory and brain activity •CCT outperformed paper-and-pencil training	•Difference in age and educational level
Zhang et al. (2019)	•Multi-domain cognitive training via tablet	27 older adults with MCI Age range: 55 years and above Twice a week/12 weeks	•Reasoning •Memory •Visuospatial skills •Language •Calculation •Attention	•Improvement in immediate memory and visuospatial memory abilities •No significant difference in neuropsychological test scores observed from baseline	•A small sample size •Inadequate training duration •Lack of control group
Barnes et al. (2009)	•Computer-based cognitive training (CCT) program developed by Posit Science Corporation (San Francisco, CA)	47 subjects with mild cognitive impairment Age mean: 74 years old 100 min/day, 5 days/week for 6 weeks	•Processing speed •Accuracy •Primary Memory •Working auditory memory	•Primary outcome of global cognitive function between the intervention and control groups not statistically significant •Effect sizes for measures of verbal learning and memory consistently favored the intervention	•Small sample size •Stimulating cognitive and physical lifestyle activities outside of intervention not controlled
Marusic et al. (2016)	•Computerized spatial navigation training (CSNT) protocol •Experiment-control groups	16 healthy men Age range: 55–65 years old 14 days training, 28-day recovery program	•Executive function •Attention •Processing speed	•Improved spatial navigation •Improved performance (fidelity), but visible also across other cognitive domains known to be associated with brain areas sub served by those that involve spatial navigation	•A small sample size •A short duration
Herrera et al. (2012)	•Programmed training exercises (visual recognition task) •Attention training task	22 older adults with amnestic MCI Age range: 65–90 years old 12 weeks	•Memory •Attention	•Improved episodic memory •Transfer effect between recognition vs. recall •Attention training with the visual focused attentional tasks improved information processing	•Parameters very frequently manipulated so that training tasks would continue to challenge each patient's abilities throughout training
Gooding et al. (2015)	•Randomized clinical trial •Computerized Cognitive Training (CCT) •Cognitive Vitality Training (CVT) •An Active Control Group (ACG)	96 male participants Age mean: ~76 years old 30 h of training/16-weeks	•Memory •Attention •Executive Function	•CVT showed significant improvement relative to ACG •No significant difference between participants of CCT and CVT	•Restricted demographics sample •Did not include measures to assess everyday functioning

**Table 3:** Commercially available programs and video games.

**References**	**Type of intervention**	**No of subjects and trial period**	**Cognitive domain focused**	**Findings**	**Limitations**
Hyer et al. (2015)	•Cognitive training program Cog Med for the intervention group and Sham for the active control group	68 older subjects with Mild Cognitive Impairment (MCI) Age range: 65 years and above 7 weeks	•Working memory	•Improved working memory of both groups •Cog Med group had higher satisfaction ratings	•A small sample size •A short-term period of the trial; a lack of the program intensity
Walton et al. (2015)	•Internet-based commercially available program- Brain trainer •Active-control group	28 healthy older subjects Age mean: 64.18 years old 28 days	•Processing speed •Memory execution function •Visuospatial memory and ability •Working memory	•Improved reaction time for both groups •Significant improvement in accuracy and spatial working memory for treatment group	•A lack of the follow up assessment •A small sample size •A short-term period
Toril et al. (2016)	•Commercially available video games Lumosity used for training •Experimental control group	39 healthy older adults Age mean: 69.95 years old (experimental group) & 73.20 years old (control group) 7–8 weeks	•Visuospatial working memory •Episodic memory •Short-term memory	•Improved visuospatial and working memory performance •Effects maintained over a 3-month no-contact follow-up period in short term memory and episodic memory	•A small sample size •Study did not evaluate the effects of training older adults with video games on everyday life tasks •A passive control group
Simon et al. (2018)	•CogMed for training participants •An active control group	82 healthy older adults from 2 countries Age range: 65 and above 5 times a week/5 weeks	•Working memory	•Improved working memory and processing speed	•Not able to conclude if cultural differences between sites affect cognitive measures •A moderate sample size •The transfer effect observed on only one cognitive task •Lacked a baseline assessment
Strenziok et al. (2013)	•Brain Fitness (BF-auditory perception) •Space Fortress (SF-visuomotor/working memory) •Rise of Nations (RON strategic reasoning)	42 healthy ‘older adults' Age not specified 6-week training session	•Auditory perception •Visuomotor working memory •Strategic reasoning	•BF training: improvement in everyday problem-solving and reasoning •SF: improvement in untrained everyday problem-solving •RON: no effect on everyday problem-solving or reasoning and reduced working memory performance	•Benefits common to all three tasks were less detectable
Peretz et al. (2011)	•CogniFit Personal Coach, •Computer games group	155 healthy older adults Age range: 61–75 years old 3 sessions/week/3-month period	•17 cognitive abilities	•Both approaches generated cognitive benefits •Conclusion: Regular mental stimulation will result in improved cognitive ability	•The ceiling effects in the measurement instrument •The lack of health and quality-of-life endpoints •The absence of a strict follow-up to monitor additional aspects of adherence •The lack of a postintervention follow-up
Finn and McDonald (2011)	•Lumosity	25 participants with MCI Age range: 60 years and above 6–8 weeks	•Attention •Processing speed •Visual memory •Cognitive control.	•Improved performance on the trained tasks over time •Improvement on a measure of visual sustained attention in treatment group •No significant changes noted on other primary outcome measures •No generalization to self-reported memory functioning or perceptions of control over memory	•A small sample size •No control group •Training at home, using own computers
Ballesteros et al. (2014)	•Lumosity	40 healthy older adults Age range: 57–80 years old 20 1 h non-action video game training sessions/10–12 weeks	•Processing speed •Attention •Executive control •Spatial working memory •Episodic memory •Subjective well-being	•Trained group showed enhancements in controlled processing, attention, immediate and delayed •Recall memory •Affection and assertiveness. •Trained participants neither showed transfer to executive control nor to spatial WM •Reduced distractibility in trainees by improving alertness and attention filtering •Marginal improvement observed in affection and assertiveness (two dimensions of subjective well-being) •No significant impact of video game training on executive functions	•A small sample size •Generalizability to everyday life tasks not examined •Additional time spent, and development of rapport/relationship, with researchers, may have effected motivation of experimental group—complicating conclusions regarding neuroplasticity
Styliadis et al. (2015)	•Posit training •Divided into five groups: three experimental groups: cognitive and/or physical training; two control groups: active and passive	70 right-handed MCI older adults Aged 60 years old and above 8 weeks	•Verbal memory •Executive functions •Independent living	•Combined interventions, occurring either sequentially or simultaneously, show promise in maintaining or improving cognitive functions •Combined training can improve general cognitive performance and subjective measures of functional status as compared to a no-treatment control •The other experimental (CT, PT) and control groups (AC) did not show significant alterations in their cortical activity after training	•Not a blind study •A small sample size
Miller et al. (2013)	•Brain Fitness •Intervention-control group	84 healthy older adults Age mean: 81.8 years old 5 days a week/20–25 min each day/8 weeks	•Short and long-term memory •Language •Visual spatial processing •Reasoning/problem solving •Calculation skills	•Improved delayed memory scores •Improved cognitive performance over extended period, including memory and language	•A small sample size •A comparatively short follow-up period of 6 months •Most participants were well-educated and Caucasian •Wide variability in number of sessions for each group •Participants not screened for MCI
Lin et al. (2016)	•INSIGHT online training program (Posit Science) •Active control group	21 older adults with MCI Age range: 60 years or above 6 weeks VSOP training	•Processing speed •Attention	•VSOP lead to improvement in trained and untrained domains like working memory	•A small sample size •The training effects not specified
Hughes et al. (2014)	•Group-based Wii interactive video gaming	20 older adults with MCI Age mean: 77.4 years old 90 min sessions/24 weeks	•Not specifically mentioned	•Older adults with MCI are capable of engaging in interactive video gaming over a period of 6 months •Community-dwelling older adults with MCI are capable of, enjoy, and are stimulated by, interactive video games	•A small sample size

### Quality Assessment

Table 1 presents an assessment of the methodological quality of the included studies. The quality assessment ranged from 3 to 7, with an average of 5.25 out of 9. All included studies have a “good” methodology. A study by Corbett et al. (2015) had the highest methodological quality. The score of item 1 was low among the included studies because most of the studies had a small sample size; below 100. All studies have clearly mentioned the age range or mean age group of their sample, except a study by Šabanović et al. (2013) which identifies their sample only as “older adults.” While all studies had intervention duration longer than 3 weeks, one study by Walton et al. (2015) was identified as having a very short duration at just 28 days.

### Participants, Sample Size, and Duration of Intervention

Among the 24 studies, all the participants were older adults. However, 15 studies had healthy older adults (Verhagen et al., 1998; Tapus et al., 2007; Finn and McDonald, 2011; Gross et al., 2011; Herrera et al., 2012; McAvinue et al., 2013; Strenziok et al., 2013; Gooding et al., 2015; Hyer et al., 2015; Walton et al., 2015; Marusic et al., 2016; Nouchi et al., 2016; Toril et al., 2016; Yeo et al., 2018)^3^, 8 studies (Peretz et al., 2011; Rose et al., 2012; Ballesteros et al., 2014; Corbett et al., 2015; Styliadis et al., 2015; Marusic et al., 2016; Simon et al., 2018; Requena and Rebok, 2019) used participants with MCI and 1 study (Zhang et al., 2019) had participants with subclinical cognitive decline. The age range of these studies varied between 55 and 90, with only 1 study (Finn and McDonald, 2011) with an age range not provided, though they did identify the participants as “older adults.” The duration of interventions was between 14 days and 6 months, with 1–3 sessions per week on an average.

### Cognitive Functions Measured

Among the 24 studies, a variety of foci were employed examining the impact on cognitive function. Some studies focused on a single function, whereas others explored two or more. The following are areas where quantitative data were gathered: processing speed, memory attention, and reasoning. Processing speed is defined as the ability to quickly process information. A total of 8 studies (Barnes et al., 2009; Finn and McDonald, 2011; Bozoki et al., 2013; Ballesteros et al., 2014; Walton et al., 2015; Lin et al., 2016; Marusic et al., 2016; Nouchi et al., 2016) measured processing speed. Results of 5 studies (Ballesteros et al., 2014; Walton et al., 2015; Lin et al., 2016; Marusic et al., 2016; Nouchi et al., 2016) showed there was improvement. Memory is the ability to retain, store, and recall information (Kueider et al., 2012). There are many different types of memory (e.g., recall, recognition, episodic, verbal, visual, and working). While most studies only examined memory as an overall cognitive function, some divided it into subcategories. Eleven studies (Barnes et al., 2009; Bozoki et al., 2013; McAvinue et al., 2013; Strenziok et al., 2013; Ballesteros et al., 2014; Hyer et al., 2015; Walton et al., 2015; Nouchi et al., 2016; Toril et al., 2016; Simon et al., 2018; Yeo et al., 2018) focused on working memory; 2 studies (Hyer et al., 2015; Simon et al., 2018) out of these had working memory as the sole cognitive domain for the study. Both (Hyer et al., 2015; Simon et al., 2018) showed significant improvement in the area of working memory; 1 study (Hyer et al., 2015) had participants with mild cognitive impairment (MCI) while the other (Simon et al., 2018) had healthy older adults. Both studies used commercially available CogMed video games in their training. The study with healthy older adults demonstrated transfer effects resulting in improvement of processing speed as well. Out of the remaining 9 studies (Barnes et al., 2009; Bozoki et al., 2013; McAvinue et al., 2013; Strenziok et al., 2013; Ballesteros et al., 2014; Walton et al., 2015; Nouchi et al., 2016; Toril et al., 2016; Yeo et al., 2018) which focused on multiple domains in addition to working memory, 3 studies (Barnes et al., 2009; Walton et al., 2015; Toril et al., 2016) showed improvement, whereas 6 studies (Bozoki et al., 2013; McAvinue et al., 2013; Strenziok et al., 2013; Ballesteros et al., 2014; Nouchi et al., 2016; Yeo et al., 2018) showed no significant change in working memory. Other categories of memory, like short-term memory and episodic memory, showed improvement after interventions. Some studies which considered memory as a single domain also showed levels of improvement. However, there was one study that showed no improvement at all (Gross et al., 2011). Attention can be understood as the process by which an individual directs or focuses on specific auditory or visual stimuli in the environment. There were 10 studies (Finn and McDonald, 2011; Herrera et al., 2012; Ballesteros et al., 2014; Corbett et al., 2015; Gooding et al., 2015; Lin et al., 2016; Marusic et al., 2016; Yeo et al., 2018; Requena and Rebok, 2019; Zhang et al., 2019) which included attention as one of the focused domains. Attention was seen to improve significantly in 8 studies (Finn and McDonald, 2011; Herrera et al., 2012; Ballesteros et al., 2014; Corbett et al., 2015; Gooding et al., 2015; Lin et al., 2016; Marusic et al., 2016; Requena and Rebok, 2019) while 2 studies (Yeo et al., 2018; Zhang et al., 2019) found no improvement. Reasoning is the action of thinking about something in a logical, sensible way. Five studies (Bozoki et al., 2013; Miller et al., 2013; Corbett et al., 2015; Nouchi et al., 2016; Zhang et al., 2019) focused on reasoning as one of the cognitive domains. While 2 studies (Bozoki et al., 2013; Nouchi et al., 2016; Zhang et al., 2019) did not find evidence of impact after from intervention, the 3 remaining studies (Miller et al., 2013; Corbett et al., 2015) found significant improvements.

### Robots Used in Elderly Care

Out of another set of 25 studies, 7 (Tanaka et al., 2012; Kim et al., 2015; Moyle et al., 2015, 2017; Soler et al., 2015; Jøranson et al., 2016; Thodberg et al., 2016) were shortlisted that implied usage of robots in elderly care (Table 4). Of these, 2 studies (Kim et al., 2015; Soler et al., 2015) examined the effect of using a robot for cognitive training with the elderly. Most of the studies were based on the usage of robots as a companion (Jøranson et al., 2016; Moyle et al., 2017) or explored their role in affective therapy (Tanaka et al., 2012; Moyle et al., 2015; Thodberg et al., 2016). However, all robotic studies yielded a positive outcome, thus making it one of the most suitable methods to impart cognitive training at home since it can be supervised by the robot. Also, the difficulty level of the program can be controlled through the robot; by maintaining and analyzing the data, and then adjusting the difficulty level of the training accordingly.

**Table 4:** Robots used in elderly care.

**References**	**Type of robot and role of robot**	**No of subjects and trial duration**	**Place of study**	**Intervention**	**Outcome**
Kim et al. (2015)	•Role: assisted in cognitive training •Robots—Silbot and Mero	85 participants Age range: 60 and above 12-week study	N/A	•Participants randomized into 3 groups: •Traditional cognitive training •Robot-assisted cognitive training •No intervention group	•Conventional cognitive training group showed less cortical thinning •Robot-assisted group showed greater results
Tanaka et al. (2012)	•Role: therapeutic •Robot–Nodding Kabochan communication robot	34 healthy female adults Age range: 66–84 years old 8 weeks study	Home	•Participants randomized into 2 groups: •Group with communicative Kabochan robot which communicated with users •Group with a control robot looked like Kabochan but did not communicate	•The experimental group slept better •Had decreased levels of saliva cortisol •Showed improved cognitive function, (executive and verbal memory function)
Jøranson et al. (2016)	•Role: companion •Robot—PARO	53 older adults with MCI or dementia Age range: 65 and above 12 weeks study	Nursing home	Participants randomized into two groups:•The intervention group with PARO—the harp seal robot •The control group which carried out treatment as before the study	•The intervention group showed improved quality of life levels but only for patients with severe dementia •No significant difference seen in quality of life levels in mild-to-moderate dementia in the intervention group as compared to control group
Thodberg et al. (2016)	•Role: affective therapy •Robot—PARO	100 participants Age mean: 85.5 years old 6 weeks	Nursing home	•Supervised interaction with a dog, PARO or a toy cat	•The dog and the robot gained more interaction than the toy cat •Over time robot interaction deceased as compared to interaction with dog •Depression scores improved over the study
Moyle et al. (2017)	•Role: companion therapy •Robot PARO	415 participants with dementia Age mean: 85 years old 10 weeks	Long-term care facilities	•One-on-one interaction with PARO •Switched on and with PARO switched off and a control group	•Participants in the PARO switched on group were more engaged verbally and visually than compared to the users in PARO switched off group •PARO switched on group had improved pleasure and reduced agitation levels
Moyle et al. (2015)	•Role: therapeutic •Robot—CuDDler	5 female participants with dementia Age mean: 84 years old 5 weeks	Nursing home	•One–to–one interaction with the CuDDler	•Agitation levels increased among the 5 participants
Soler et al. (2015)	•Role: cognitive and physical therapy •Robots—NAO & PARO	Phase 1−101 participants with dementia Age mean: 84.7 years old 3 months Phase 2−110 participants with dementia Age mean: 84.7 years old 3 months	Nursing Home	•Phase 1–supervised cognitive, musical, and physical group therapy with NAO •Phase 2–supervised cognitive, musical, and physical group therapy with PARO	Phase 1: •Decreased apathy in both the groups •Increased delusion in the NAO group •Increased irritability in both groups Phase 2: •Increased hallucinations and irritability in both the groups

## Discussion

This review first summarizes the types of CCT programs that have been employed for improving cognitive function or attenuating cognitive decline in both healthy older adults and older adults with MCI. It also examines the impact of these programs. Based on this review, CCT appears very promising as a tool to improve the cognitive abilities of healthy older adults and adults with MCI who have a higher risk of acquiring dementia or Alzheimer's disease. Timely training may prolong the onset of dementia and Alzheimer's disease; still, there are a few concerns to be discussed in this section. This review next presents, how robots have been used in elderly care to ease their living.

In our shortlisted studies, the cognitive training can be categorized as self-designed cognitive training, custom-made for the program (Barnes et al., 2009; Herrera et al., 2012; Rose et al., 2012; Bozoki et al., 2013; McAvinue et al., 2013; Corbett et al., 2015; Gooding et al., 2015; Marusic et al., 2016; Nouchi et al., 2016; Yeo et al., 2018; Requena and Rebok, 2019; Zhang et al., 2019), and as commercially-available training programs and video games (Finn and McDonald, 2011; Peretz et al., 2011; Miller et al., 2013; Strenziok et al., 2013; Ballesteros et al., 2014; Hughes et al., 2014; Hyer et al., 2015; Styliadis et al., 2015; Walton et al., 2015; Lin et al., 2016; Toril et al., 2016; Simon et al., 2018). Based on this review, self-designed cognitive training interventions demonstrated an improvement in processing speed, working memory, executive function, visual spatial ability, and attention. For example, in one of the studies; Corbett et al. (2015), an online 6-month randomized 3-arm controlled trial was conducted. The study compared general cognitive training (GCT), evidence-based reasoning and problem-solving cognitive training (ReaCT) and a control group. ReaCT focused on 3 reasoning and 3 problem solving tasks and GCT involved cognitive tasks covering mathematics, attention, memory and visuospatial ability. Participants were asked to undertake these training for 10 min daily. As participants improved the task difficulty increased to maintain the challenge and improve performance. The control group performed equivalent internet-based tasks involving a game in which people were asked to put a series of statements in correct numerical order. This trial showed that there was considerable improvement in all the cognitive domains mentioned above.

Commercially available trainings like Cogmed and Lumosity improved visuo-spatial functions, episodic memory, working memory and attention as these commercially available programs have designed games in a manner which helps in improving the above-mentioned cognitive domains. For example, Hyer et al. (2015) used Cogmed for their study, which had participants with MCI, the study focused on improvement of working memory. Twenty-five sessions were conducted in 5–7 weeks for 40 min per day, where the participants were given exercises, that involved the temporary storage and manipulation of sequential visuospatial and/or verbal information. Each participant had a coach who ensured the proper completion of tasks in a timely manner. Improvement in working memory of participants was seen after the training (Hyer et al., 2015). Video game-based training had a significant impact on measures of reaction time and processing speed but were not very impactful on executive function or memory (Ballesteros et al., 2014). For example, Ballesteros et al. (2014) in their study used non-action video games for training, 50 healthy older adults for 20–1 h sessions for 12 weeks. They observed enhancements in controlled processing and attention but no significant improvement in working memory and executive functions. Consequently, interventions based solely on video games may not be, strictly speaking, of significant benefit to improve cognitive function.

In addition, most studies used a small sample size, and duration of training was short. Many did not carry out follow-up checks after training to ascertain the existence of long-term effects. One of the studies (Lampit et al., 2014) indicated that CCT should be done for a minimum of 30 min because synaptic plasticity is only possible after 30–60 min of stimulation. It also noted that training sessions should not exceed three sessions per week, otherwise the training appeared to produce the opposite of the intervention objective (Lampit et al., 2014). This observation could be utilized for designing a program with methodology that produces the best outcome, Researchers also claim that computer-based cognitive training has moderate effects in improving cognitive functioning in healthy older individuals, but the training's effectiveness varies across cognitive domains and is determined by design choices.

A very important element regarding the efficacy of cognitive training is the question of transfer effect, which is explained in the introduction section above. Even though the above statements raise concerns regarding the efficacy of these trainings, research is still in progress to develop CCT programs for elderly as clinical studies show that these trainings may generate meaningful transfer effects (Bozoki et al., 2013). The reviewed studies show that the transfer effect of cognitive training to untrained tasks is mixed; some trainings have achieved far transfer and some have not. This is not to say that targeted training did not improve specific cognitive measurements. It was seen that some tasks improve cognitive performance, but without transfer to untrained tasks.

Another issue which may produce hindrance for cognitive benefits is allowing participants the freedom to choose which games they play and the option to set the levels of difficulty according to their personal preference (Bozoki et al., 2013). Importantly, the findings indicate that participants tend to select options with the least challenge. This produces diminished cognitive benefits since the resulting focus of training is often limited to fewer cognitive domains. While some CCT can be conducted from home, which is more convenient for those home-bound, and allows users to work at their own pace and to focus more on the areas that need improvement, there are a few points to consider. For CCT to produce long-term benefits it needs to be performed for a significant duration; additionally, it should be rigorous, repetitive, and consistently challenging (Klimova, 2016). The training to be effective at home must be supervised with a specialized trainer/care giver, if executed in an impromptu manner it may not yield the sought-after results, as stated above. Thus, even after certain benefits, the need of a trainer persists.

There are additional issues concerning the development of CCT programs for the elderly. Older individuals, for example, may not possess the required interest to use computer programs—though most do not require the user to be tech-savvy, and basic instructions are given prior to the start of the program. There may also be the need to develop age-specific (Wolfson and Kraiger, 2014) and culture-specific computer-based training programs and formats. Interestingly, little is known about the impact cultural background can have on this form of training. Since culture is a way of social life for people, it influences lifestyle, personal identity and one's relationship with others (Bruno et al., 2017). A cultural innovation can trigger changes in general cognitive capabilities (Bender, 2019). It has its share of effect on cognitive skills and information processing, because of this, a consideration of user culture when designing a robot would ensure older users are more comfortable and motivated. The use of language is one of the key abilities that contributes to our existence, it facilitates cooperation and allows us to agree on values and norms, making the foundation of our communities (Bender, 2019). If a program is culture specific and is designed in the native language of the user, they may relate more, especially elder people; as everyone may not be familiar with the standard English language and would understand the instructions better in their native language. Also, if the program is designed based on their local culture values and traditions, they may connect more to the exercise. This, in turn would help them complete the tasks assigned, without leaving the study mid-session. Another concern, possibly related, is that many users may not find it sufficiently engaging or motivating to carry out the trainings via a PC or a tablet, whereas the presence of a physical entity in the form of a social robot may more strongly compel users to complete the trainings since the robot can appraise, remind and encourage users to carry out the trainings.

While most studies suggest cognitive training works best when done for a longer duration, and computer-based cognitive training makes it easier for participants to receive this training since it is from the comfort of their homes, there is still the requirement of a specialized trainer/care-giver to monitor improvement and adherence to learner best practice. Also, specialized therapists need to be present to guide users through their execution, to continuously challenge them as they master a particular level of a program and to provide feedback during the task. Specialized therapists, additionally, need to keep track of performance to later draw a conclusion and maintain a progress report over a period of time (Broekens et al., 2000).

Most of the participants in our studies were above 60 (total 3,270 participants; 7.7% with MCI), except for the patients in three studies (Ballesteros et al., 2014; Marusic et al., 2016; Zhang et al., 2019), where participants were 55 and above years of age (total 83 participants, 32.5% with MCI). If the sample age is divided into two sections as young older adults (below 75 years) and old-older adults (above 75 years), only 3 studies (Miller et al., 2013; Gooding et al., 2015; Lin et al., 2016) have specified that the sample was above 75 (total of 200 participants, 10% with MCI). However, on analyzing the outcomes, there were no specific effects seen on CCT because of age difference. It can be said that CCT has a generalized positive effect for elderly (aged above 55 years) and is not age dependent.

It can be summarized that the CCT is mostly beneficial and shows improvement in older adults. Issues related to training location need to be addressed; either it is performed at a facility, or at home. When done at the former, the participant is required to attend regularly. This may be a demotivating factor for some since all participants may not have easy access to transportation and the commute may be a challenge. This challenge may be further compounded by, the additional cost of public or private paid transports. When performed at home it is usually unsupervised and, as a consequence, the participant carries out the training in a sequence according to their own choice of activity. This may result ineffective engagement, since it is highly plausible participants will choose exercises that require less than optimal effort, and so limit the potential cognitive benefits.

Robots, however, have come a long way and are now available widely to assist in numerous ways. Human-robot interaction (HRI) has improved meaningfully over the past decade and is a technology being used in the healthcare industry in the form of socially assistive robots (SAR). SARs have proven to have immense potential in elderly care, promising to reform its delivery. In this review the existing studies have been extensively searched and the following roles of SAR can be outlined broadly as: (1) Companion Robots—the function of these robots is to provide companionship and alleviate anxiety and loneliness. They are commonly designed in animal-like forms providing companionship as a pet would, without the overhead of animal care. (2) Care Robots—these are designed to assist the elderly in their daily activities, for example reminding them to take their medicine on time, connecting them with their loved ones through voice or video calls, detecting falls, and notifying the appropriate authority in the case of an emergency. Some are even designed to fetch things from other rooms etc. They are very suitable, in fact, for elderly people living alone or people who have difficulty performing certain movements. (3) Therapy Robots—they are designed to carry out therapy sessions, whether physical exercises, or cognitive training. With this objective, they monitor the improvements of its users, intelligently adjusting the level of difficulty of exercises when needed to place them specifically within the range of proximal development, and to assess user mood, interest, and level of engagement with a variety of sensors and programs which a simple computerized cognitive training program is unable to achieve. The additional information gathered by the robot creates a qualitatively superior, and hence, more meaningful interaction for the user. Furthermore, the appearance of a robot can be altered to produce a pet robot or humanoid; likewise, a robot can be programmed to mimic enthusiasm and other emotions, to possess an appealing voice or the preferred gender. These elements enhance communication considerably. Because of this, in contrast to the effectiveness of tablets and CCT programs, where robotic intervention excels is in its pronounced capacity to engage and therefore motivate users to complete exercises and succeed in cognitive training. Research has shown that users are able to develop an emotional connection and a virtual relationship with their robots, helping to produce the superior social, emotional and cognitive benefits of the intervention, and raise quality of life scores (Gazzola et al., 2007; Šabanović et al., 2013; Liang et al., 2017; Bender, 2019).

Still, there are, in fact, few studies that use robotics exclusively as a medium for cognitive training. There is ample opportunity to do extensive research in the development of assistive robots for cognitive training, in particular, developing culturally appropriate robots for maximum benefit to the user. In contrast to conventional CCT programs, we believe the use of robots for cognitive training of elderly individuals experiencing age-related cognitive decline may produce significantly greater positive impact. For example, Kim et al. (2015) used both cognitive training and robot-assisted training in their study, they found the robot assisted training to be more effective.

There are a few limitations that require further research. Firstly, future trials should aim for a larger sample size and a longer intervention duration; additionally, they must directly compare the different alternatives of training to identify the most effective. Secondly, studies focusing on multiple cognitive domains should have greater organization when carrying out activities on specific domains. This would make it easier to conclude which domains have benefited the most. Thirdly, hardly any studies are available which show culturally appropriate cognitive training approaches. Future research which consider this may produce improved cognitive function. Fourthly, many studies have inconsistent research designs making it difficult to extract a clear and crisp conclusion. This should be considered. Also, only studies published in English were included, other language studies may have more insights, but were excluded as it would be difficult to understand and interpret them correctly for this review; which could have resulted in a biased outcome. A final limitation seen is the absence of research which addresses the needs of a minimally educated elderly population. We should aim to develop programs that are able to cater to people with basic levels of education, or no education at all. Ultimately, this would allow these programs to have a wider reach, since many countries around the world may have an elderly population that is not highly educated or literate.

## Conclusion

Based on this review, we can conclude that computerized cognitive training targeting healthy older adults and adults with MCI is moderately beneficial in improving various cognitive functions. Various methods of available trainings have yielded improved working memory, attention, processing speed, episodic memory, visuo-spatial functions, executive functions; however, there are a few issues with CCT which can be overcome by the introduction of SARs in this field.

Future studies will need to focus more closely on the key psychological, cultural and socio-economic factors of its participants. Researchers should double their efforts to identify the key-mechanisms for improving the cognitive and everyday functions of elderly. Large sample, longer-duration experiments are needed with a control group preferably to get a more generalized outcome. Furthermore, cultural considerations, as mentioned, need to be made by developers to strengthen acceptance and engagement by users. Considerations, such as language and lifestyle, would help reach a more wide-ranging population. Finally, most of the present examples of SARs are designed to provide companionship or assistance to the elderly in their daily activities. Future research should aim to make a SAR, not just culturally oriented in its disposition, but able to carry out the much needed cognitive trainings with the elderly in a highly organized manner targeting multiple domains, while maintaining the results and scores and adapting the program to make it constantly challenging and fun for its user. All of this would result in greater success and produce opportunities for smart aging.

## Author Contributions

FA, SK, AV, SS, RN, and RK contributed conception and design of the study. FA, SS, AV, and RN prepared the materials. FA, SK, and AV wrote the manuscript. SS, RN, and RK reviewed the manuscript and gave comments. All authors contributed to the final manuscript revision, read and approved the submitted version.

### Conflict of Interest

The authors declare that the research was conducted in the absence of any commercial or financial relationships that could be construed as a potential conflict of interest.

## Footnotes

^1^United Nations: World Population Prospects The 2015 Revisions (2015). Available online at: http://esa.un.org/unpd/wpp/Publications/Files/Key_Findings_WPP_2015.pdf (accessed September 2015).

^2^Dementia Statistics, Alzeihmer's Disease International. Available online at: https://www.alz.co.uk/research/statistics.

^3^*Introduction: Human – Robot Interaction*. Available online at: https://humanrobotinteraction.org/1-introduction/.
